# Dielectric Characteristics, Electrical Conductivity and Solvation of Ions in Electrolyte Solutions

**DOI:** 10.3390/ma14195617

**Published:** 2021-09-27

**Authors:** Vladimir V. Shcherbakov, Yuliya M. Artemkina, Irina A. Akimova, Irina M. Artemkina

**Affiliations:** Department of General and Inorganic Chemistry, Mendeleev University of Chemical Technology, Miusskaya sq. 9, 125047 Moscow, Russia; yulyart@muctr.ru (Y.M.A.); akimosha1@yandex.ru (I.A.A.); irinartemkina@mail.ru (I.M.A.)

**Keywords:** electrolyte solutions, ion solvation, electrical conductivity, dielectric constant, distance between ions in solutions

## Abstract

Solvation and association of ions in solutions largely depend on the dielectric properties of the solvent, the distance between ions in solutions, and temperature. This paper considers the effect of temperature on static dielectric constant (DC), dipole dielectric relaxation (DR) time, and limiting (ultimate) high frequency (HF) electrical conductivity (EC) of water and some polar solvents. In the investigated temperature range (0–370 °C), the static DC and DR time of water decrease, and limiting HF EC passes through a maximum at 250–300 °C with temperature growth. The dielectric characteristics of methanol, ethanol, and propanol behave in a similar way. It is shown that the existence of an HF EC temperature maximum is due to the different nature of the temperature dependences of DC and DR time. It is suggested that the same dependences are responsible for the presence of a maximum in the temperature dependences of the dissociation degree and the ionic product of water. The influence of non-electrolytes concentration as well as metal salts on the dielectric properties of their aqueous solutions is considered. The limiting HF EC of water determines the specific EC value of aqueous electrolyte solutions. Analysis of the absorption of microwave energy by polar solvents, as well as aqueous solutions of non-electrolytes and electrolytes, at a frequency of 2455 MHz is carried out. The optimal conditions for high-frequency heating of solutions have been established. The distance between ions in aqueous solutions of inorganic salts and in non-aqueous solutions of ionic liquids is calculated. It is shown that the maximum on the concentration dependence of the specific EC can be related to ions association.

## 1. Introduction

Electrolyte solutions hold a special place in chemical science [1,2,3,4]. They play an important role in chemical technology since most chemical reactions in industry take place in solutions [5]. Most biological processes also take place in solutions containing electrolyte ions [6]. Despite a significant number of works devoted to the study of electrolyte solutions containing metal ions, the solvation of these ions in electrolyte solutions has not been adequately studied. At present, the structure of electrolyte solutions has not been finally determined [3]. It is not clear how it changes in going from dilute solutions to concentrated solutions and how the solvation of metal ions in solutions changes [4].

The properties of solutions are largely determined by interactions between solute and solvent molecules, and in the case of electrolyte solutions, interactions between ions, between ions and a solvent, as well as between undissociated electrolyte molecules, solvent, and ions. All of these interactions are largely electrostatic in nature. The strength of these interactions is determined by the dielectric characteristics of the medium in which the ions and dipole molecules are located, as well as the distance between ions and molecules in solutions [1]. Therefore, in this work, special attention is paid to consideration of the dielectric properties of polar solvents in a wide range of temperatures and the dielectric properties of solutions in a wide range of concentrations, as well as estimation of the distances between particles (ions and molecules) in solutions. The change in these properties in going from dilute solutions to concentrated ones is considered.

The most important property of electrolyte solutions is their electrical conductivity (EC). This paper considers the concentration and temperature dependence of the specific EC of aqueous solutions of electrolytes and some solutions of ionic liquids in acetonitrile, dimethyl sulfoxide (DMSO), and dimethylformamide (DMF). A relationship is established between the specific EC of solutions and the dielectric properties of polar solvents [7].

## 2. Dielectric Characteristics of Polar Solvents

The most important dielectric characteristics of electrolyte solutions are the static dielectric constant (DC) ε_s_, the time of dipole dielectric relaxation (DR) τ, and the limiting high-frequency (HF) electrical conductivity (EC) κ_∞_, calculated on the basis of these values. The limiting HF EC is determined by the ratio of the absolute DC to the DR time [7]:

(1) $$\kappa_{\infty }=\frac{\varepsilon_{s}\varepsilon_{0}}{\tau }$$

In this expression, ε_0_ is the absolute DC of the vacuum (ε_0_ = 8.854·10^−12^ F/m). The physical meaning of κ_∞_ is discussed below.

Let us consider the dielectric properties of polar solvents, since it is DC that determines the dissociation of electrolytes into ions in solutions. The DC of a polar solvent depends on the frequency of the electromagnetic field at which it is measured, its nature, and the temperature.

The dependence of the active ε′ and reactive ε″ components of the complex permittivity of polar solvents on the frequency ω (ω = 2π*F*) of the electromagnetic field in the simplest case is described by the Debye equations [8]:

(2) $$\varepsilon \prime =\varepsilon_{\infty }+\frac{\varepsilon_{s}-\varepsilon_{\infty }}{{1+(\omega \tau )}^{2}}$$

(3) $$\varepsilon \prime \prime =\frac{\varepsilon_{s}-\varepsilon_{\infty }}{{1+(\omega \tau )}^{2}}\times (\omega \tau )$$

At low frequencies of the electromagnetic field ωτ << 1, the polar molecules of the solvent have time to follow the change in the external electromagnetic field, and the DC of the solvent retains its constant value equal to ε_s_. At high frequencies in the range of 0.1 ≤ ωτ ≤ 10, polar solvent molecules no longer have time to follow the change in the external electromagnetic field. In this case, the active component of the DC ε′ decreases from the value of the static DC ε_s_ to its optical value ε_∞_ under the condition ωτ >> 1 (ε_∞_ ≈ *n*^2^, *n* is the refractive index). The reactive component of the complex DC ε″ passes through a maximum with increasing frequency. The maximum value of ε″ takes place under the condition ωτ = 1 [8].

The fact that the dispersion of the permittivity of water is well described by the relatively simple Debye Equations (2) and (3) is surprising to scientists [9,10], since Debye’s theory [8] is valid for dielectrics whose molecules do not interact with each other and, naturally, do not form intermolecular hydrogen bonds, as is the case in liquid water. Nevertheless, Debye’s theory [8] is in good agreement with experiment and is widely used to interpret the results of dielectric studies of polar solvents, which are given in reference books [11,12].

The frequency-dependence of the electromagnetic properties of polar solvents can be described not only in terms of the complex permittivity ε* = ε′ − *i*ε″ ($i=\sqrt{-1}$), but also in terms of the complex electrical conductivity κ* = κ′ + *i*κ″. The complex parameters κ* and ε* are summarized by the following relations [13]:

(4) $$\kappa *=i{\omega \varepsilon }_{0}\varepsilon *={\omega \varepsilon }_{0}\varepsilon \prime \prime +i{\omega \varepsilon }_{0}\varepsilon \prime =\kappa \prime +i\kappa \prime \prime$$

Substituting the Debye Equations (2) and (3) for ε′ and ε″ into Equation (4), we obtain for the active κ′ and reactive κ″ components of the complex EC of the polar solvent, taking into account Equation (1):

(5) $$\kappa \prime =\frac{\kappa_{\infty }}{{1+(\omega \tau )}^{2}}\times {\left(\omega \tau \right)}^{2}$$

(6) $${\kappa \prime \prime =\omega \varepsilon }_{0}\varepsilon_{\infty }+\frac{\kappa_{\infty }}{{1+(\omega \tau )}^{2}}\times (\omega \tau )$$

Equations (5) and (6) describe the dispersion of high-frequency EC of polar solvents. The active high-frequency (HF) EC of the polar solvent κ′ increases with increasing frequency and, under the condition ωτ >> 1, reaches its maximum value κ_∞_, which does not depend on the frequency and is determined by Equation (1). It can be said that at high frequencies, polar solvents become conductors and their HF EC exceeds the specific EC of solutions of inorganic salts in these solvents. The dispersion of HF EC of polar solvents and electrolyte solutions is described in [14,15].

The κ_∞_ value is a fundamental characteristic of a polar solvent. As will be shown below, the limiting HF EC of a polar solvent determines the temperature dependence of the specific EC of solutions of inorganic metal salts in polar solvents. In [14,15], the value κ_∞_ was proposed to be called the limiting HF EC of a polar solvent.

The values of the static DC ε_s_, the time of dipole dielectric relaxation τ, and the limiting HF EC κ_∞_ of some polar solvents are given in Table 1.

The static DC of a polar solvent can be considered as its structural characteristic. An abnormally high DC value occurs for protic solvents with hydrogen bonds (water, formamide, N-methylformamide), as seen in Table 1. In aprotic solvents, the ε_s_ value is also related to their structure. Acetonitrile (AN) and dimethyl sulfoxide (DMSO), for example, have approximately the same dipole moment μ ≈ 4D. Acetonitrile contains 25% more dipole molecules per unit volume than DMSO (molar volumes ratio *V*_m_^ACN^/*V*_m_^DMSO^ 0.75, at 20 °C [16]), and AN must have a higher dielectric constant than DMSO. In fact, ε_s_(DMSO) is 11 units higher than ε_s_(AN), since there is practically no dipole–dipole interaction in DMSO, while in AN there is an antiparallel arrangement of neighboring dipoles [16].

The dipole dielectric relaxation time τ, characterizing the mobility of polar solvent molecules, can be considered as its kinetic characteristic. The smaller the value of τ, the more mobile the solvent molecules. Abnormally high mobility is characteristic of acetone and acetonitrile molecules (Table 1). The mobility of polar solvent molecules decreases with an increase in their size: with an increase in the length of the hydrocarbon radical, when passing from methanol to butanol, the mobility of an alcohol molecule decreases by almost 10 times (Table 1).

The limiting HF EC is proportional to the ε_s_/τ ratio and combines the structural and kinetic characteristics of a polar solvent. In Table 1, polar solvents are arranged in decreasing order of κ_∞_. Water has a maximum value of κ_∞_. Water is characterized by a relatively high static DC ε_s_ and a relatively low relaxation time τ. Despite the fact that formamide and N-methylformamide are characterized by abnormally high values of static DC, their limiting HF EC is not high, which is explained by the low mobility of the molecules of these solvents. Acetonitrile and acetone occupy the second and third places in terms of the limiting HF EC. The molecules of these solvents have abnormally high mobility (Table 1). It is known that the conductivity of electrolytes in acetonitrile and in acetone solutions is anomalously high compared to other solvents [17,18].

Let us consider the effect of temperature on the dielectric characteristics of polar solvents. An increase in temperature leads to destruction of the structure of the polar solvent. Therefore, an increase in temperature leads to a decrease in its static DC [11,12]. The kinetic energy of solvent molecules increases with increasing temperature; therefore, a decrease of dipole dielectric relaxation time upon heating is observed [11,12]. Let us consider how the values of ε_s_, τ, and κ_∞_ of water and some polar solvents change specifically with increasing temperature.

The dielectric characteristics of water have been measured and calculated in a wide range of temperatures and pressures [11,12,14,19,20,21,22]. In the temperature range 0–374 °C, the static DC of liquid water monotonically decreases from 88–92 to 10–20 units, depending on the pressure (in the pressure range 1 ≤ *p* ≤ 1000 bar). It should be noted that, in contrast to the ε_s_ values, sufficiently accurate and reliable values of water τ were obtained in the temperature ranges of –4–60 °C [20] and 0–260 °C [21]. Therefore, to determine the values of τ of water at t > 260 °C in [7], the Debye equation [8] was used for the dipole relaxation time of a polar molecule τ, which has the form of a sphere of radius *a* and is located in a medium with viscosity η:

(7) $$\tau =\frac{4\pi a^{3}\eta }{kT}=K\frac{\eta }{T}$$

(*k* is the Boltzmann constant, *T* is the absolute temperature).

**Figure 1:**
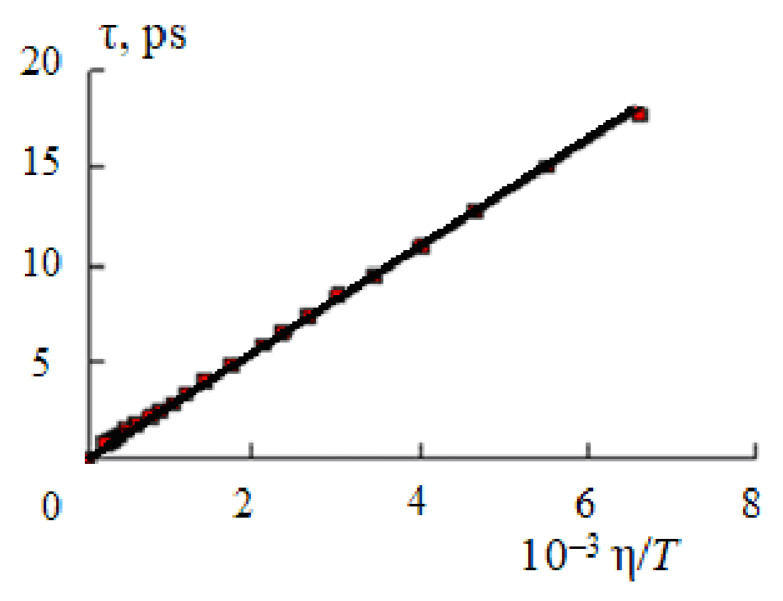
Plot of the dipole dielectric relaxation time of water τ [20,21] against the ratio η/*T* in the temperature range 0–260 °C.

Figure 1 shows the dependence of the time of the dipole dielectric relaxation of water on the ratio η/*T* (viscosity is expressed in mPa·s, temperature—K) calculated from the data of [20,21].

The value of the coefficient *K* in Equation (7) is (2.71 ± 0.04)·10^−9^ [7]. As a result of using Equation (7) to estimate the relaxation time at *T* > 260 °C in the temperature range 0–374 °C and in the pressure range 1 ≤ *p* ≤ 1000 bar, the time of dipole dielectric relaxation of liquid water also monotonically decreases from 17–18 up to 0.3–0.4 ps [11,12,20,21,22].

**Figure 2:**
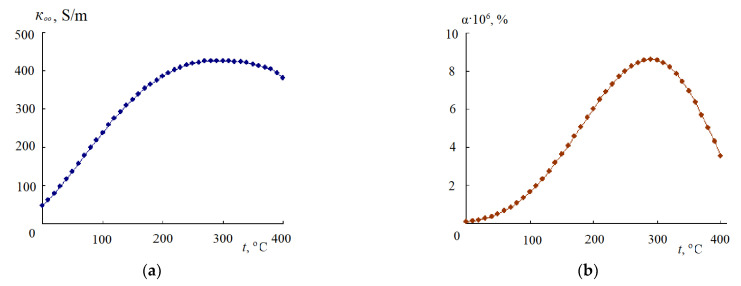
Temperature dependences of the limiting high-frequency conductivity (**a**) and degree of dissociation (**b**) of water; *p* = 500 bar.

In contrast to the monotonic decrease in the static permittivity ε_s_ and the dielectric relaxation time τ with increasing temperature, the limiting HF EC of water κ_∞_ passes through a maximum with increasing temperature [8,14] (Figure 2a).

The existence of a maximum in the temperature dependence of κ_∞_ is associated with the different nature of the dependence of the relative temperature coefficients DC ($\beta_{\varepsilon }=\frac{1}{\varepsilon_{s}}\times \frac{d\varepsilon_{s}}{dt}$) and time of DR ($\beta_{\tau }=\frac{1}{\tau }\times \frac{d\tau }{dt}$) on temperature: $\beta_{\varepsilon }=\frac{1}{\varepsilon_{s}}\times \frac{d\varepsilon_{s}}{dt}$ increases, and $\beta_{\tau }=\frac{1}{\tau }\times \frac{d\tau }{dt}$ decreases with increasing temperature. In this case, at the maximum of the limiting HF EC $\frac{1}{\varepsilon_{s}}\times \frac{d\varepsilon_{s}}{dt}=\frac{1}{\tau }\times \frac{d\tau }{dt}$ [7].

Depending on the pressure, the maximum value of κ_∞_ is observed in the temperature range 250–300 °C. At the same time, with an increase in pressure from 50 to 1000 bar, the maximum value of κ_∞_ shifts towards higher temperatures.

It is important to note that as the temperature rises, the ionic product of water *K*_w_ and the value of the degree of dissociation α of water calculated on the basis of *K*_w_ pass through a maximum in the indicated temperature range (250–300 °C) [23] (Figure 2b). Since the limiting HF EC of water is determined by the ratio of its dielectric characteristics, Equation (1), it can be assumed that the temperature maximum *K*_w_ has the same nature as the temperature maximum κ_∞_ and is observed when the relative temperature coefficients of the permittivity and dielectric relaxation time are equal.

Based on the temperature dependences of ε_s_ and τ, the existence of a temperature maximum in the degree of dissociation α of water (Figure 2b) can be explained as follows. With increasing temperature, the dielectric relaxation time decreases, and the mobility of water molecules increases. In this case, the kinetic energy of H_2_O molecules also increases and their dissociation increases in the temperature range, in which the relative temperature coefficient $\frac{1}{\tau }\times \frac{d\tau }{dt}$ is greater than the relative temperature coefficient $\frac{1}{\varepsilon_{s}}\times \frac{d\varepsilon_{s}}{dt}$. An increase in temperature also leads to a decrease in the dielectric constant of water ε_s_ and to an increase in the interaction of solvated H^+^ and OH^−^ ions and their association. The association is sharply enhanced under the condition $\frac{1}{\varepsilon_{s}}\times \frac{d\varepsilon_{s}}{dt}>\frac{1}{\tau }\times \frac{d\tau }{dt}$. A maximum also appears in the temperature dependences of α (Figure 2b) and *K*_w_.

It should also be noted that the specific low-frequency EC of water [24], as well as aqueous solutions of inorganic salts [25,26], pass through a maximum in this temperature range. A possible explanation for these dependencies will be given below.

**Figure 3:**
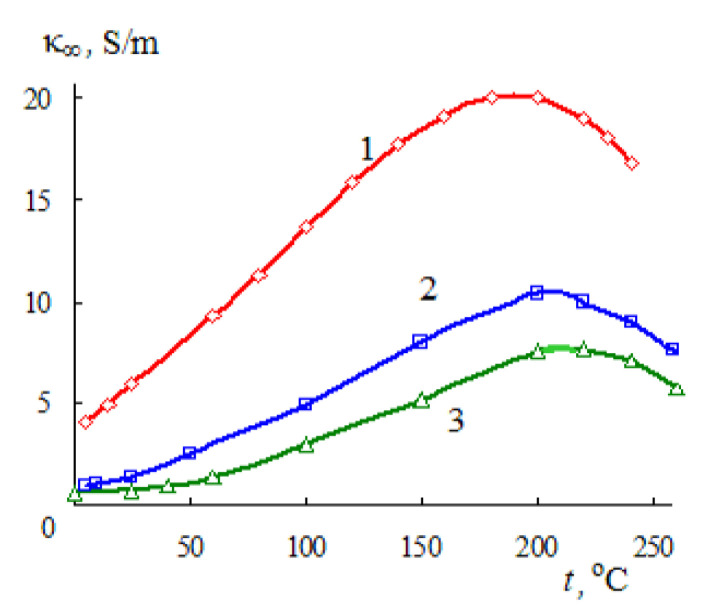
Temperature dependences of the limiting high-frequency conductivity of methanol (1), ethanol (2), and propanol-1 (3); at temperatures above the boiling point of the solvents, results are given along the liquid–vapor coexistence curve.

The dielectric characteristics of non-aqueous and mixed aqueous-organic solvents have been studied to a lesser extent and, as a rule, in a narrow temperature range [11,12]; therefore, in this work, we will consider the dielectric characteristics (ε_s_, τ, and κ_∞_) of methanol, ethanol, and propanol in a wide range of temperatures [27,28]. The temperature dependences of ε_s_, τ, and κ_∞_ of alcohols are similar to those considered above for water. Based on the values of ε_s_ and τ given in [27,28], the dependence of κ_∞_ on temperature was established [7] (Figure 3).

As in the case of water (Figure 2a), the limiting HF EC of alcohols passes through a maximum with increasing temperature. When passing from methanol to propanol, a shift of the position of the maximum towards higher temperatures is observed (Figure 3).

The dielectric characteristics of acetonitrile, dimethylformamide, and dimethyl sulfoxide and their aqueous solutions are considered below when analyzing the absorption of microwave energy by a solution and the temperature dependences of the specific EC of nonaqueous solutions of ionic liquids.

Concluding the consideration of the dielectric characteristics of polar solvents, it should be noted that they are dielectrics only at low frequencies of the electromagnetic field. Their specific EC increases with increasing frequency, and in the region of dipole dielectric relaxation, polar solvents become conductors. Under the condition ωτ >> 1, the high-frequency electrical conductivity of polar solvents reaches its maximum limiting value, which is independent of frequency and is determined by the ratio of static DC to time DR, as in Equation (1). The high-frequency EC of polar solvents determines the absorption of microwave energy by solutions and the temperature dependence of the low-frequency EC of electrolyte solutions and ionic liquids.

## 3. Dielectric Characteristics of Electrolyte Solutions

To explain the influence of the concentration of the electrolyte solution on the DC in the first half of the last century, it was proposed to consider two effects. Relaxation of ionic atmospheres [29] explained the increase in DC with increasing concentration. According to the dielectric saturation model [30], the DC should have decreased with increasing concentration.

According to the Debye–Falkenhagen theory of relaxation of ionic atmospheres [29], the dielectric constant of the solution ε_sol_ should increase in proportion to the square root of the concentration *c*:

(8) $$\varepsilon_{\mathrm{sol}}=\varepsilon_{{(H}_{2}O)}+A\sqrt{c}$$

In Equation (8), *A* is a coefficient depending on the type of electrolyte: *A*(I–I) = 3.79, *A*(I–II) = 10.9, *A*(I–III) = 13.8 and *A*(II–II) = 30.3. As a result, for DC of 0.01 M KCl and MgSO_4_ solutions, we obtain at *t* = 25 °C: ε_sol_(KCl) = 78.63; ε_sol_(MgSO_4_) = 81.30.

Measurements of the DC of electrolyte solutions, performed in the first half of the last century, in the opinion of Falkenhagen, confirmed an increase in DC with an increase in the electrolyte concentration [31]. These data, however, were obtained in the radio frequency range. When processing the experimental results, the influence of the EC on the measurement results was not taken into account [14]. The influence of incorrect accounting of specific electrical conductivity (κ) leads to an increase of the measured DC of the solution.

As a result of the analysis of the equivalent electric capacity of the electrolyte solution carried out in [32], the measured equivalent electric capacity of the *C*_e_ solution depends on the ratio of the conduction currents to the displacement currents:

(9) $$\omega CR=\frac{{\omega \varepsilon \varepsilon }_{0}}{\kappa }$$

This is determined by the expression [32]:

(10) $$C_{e}=C+\frac{C}{{\left(\omega CR\right)}^{2}}$$

In Equations (9) and (10), *C* and *R* are the electrical capacity and solution resistance, respectively.

From Equation (10), it follows that the measured capacity *C*_e_ will be equal to the capacity of the solution *C* only under the condition ω*CR* >> 1. For a 0.1 M KCl solution, for example, ω*CR* = 1 at a frequency of 3·10^8^ Hz. At this frequency, from Equation (10) we obtain: *C*_e_ = 2*C*; that is, the measured capacity *C*_e_ turns out to be twice the true capacity of the solution *C*. This means that the measured DC of the solution ε_mes_ will be twice its true value ε_sol_.

Equation (10) also implies that the measured value of the DC ε_mes_ increases in direct proportion to the square of the specific EC, if the condition ω*CR* >> 1 is not satisfied:

(11) $$\varepsilon_{\mathrm{mes}}=\varepsilon_{\mathrm{sol}}+A\kappa^{2}$$

In Equation (11), $A=\frac{1}{\omega^{2}\varepsilon_{\mathrm{sol}}}$.

**Figure 4:**
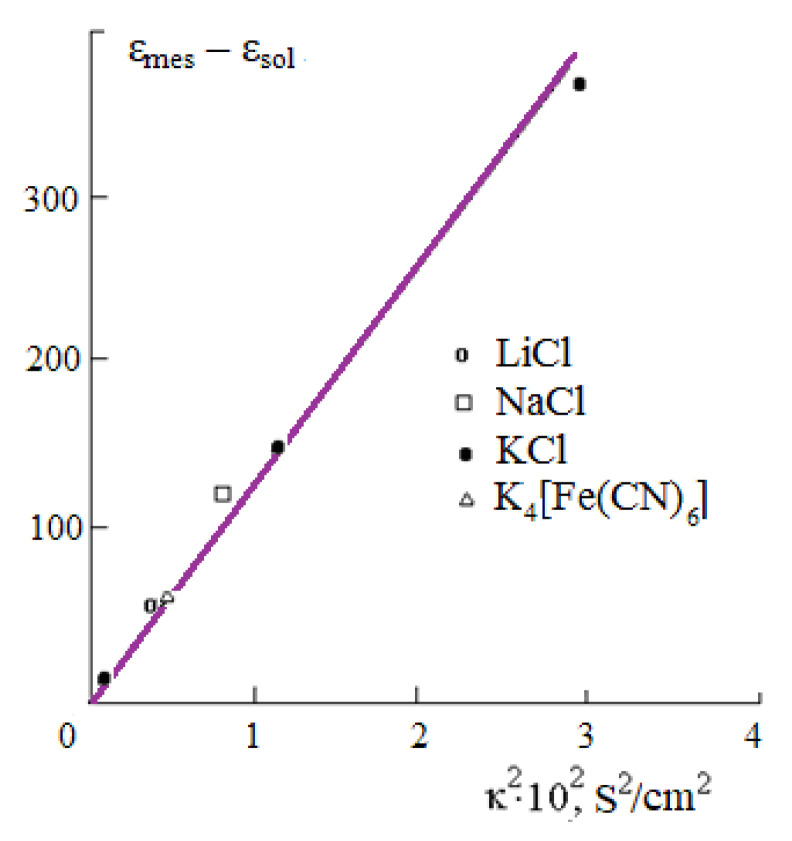
Plot of $\varepsilon_{\mathrm{mes}}-\varepsilon_{\mathrm{sol}}$ against $\kappa^{2}$ of aqueous solutions.

Analysis of the results of measurements of the DC of electrolyte solutions in the radio frequency range, given in [31], shows that the effect of electrical conductivity was not taken into account when measuring the DC. Equation (11) can be written as:

(12) $$\varepsilon_{\mathrm{mes}}-\varepsilon_{\mathrm{sol}}=A\kappa^{2}$$

whence it follows that the difference $\varepsilon_{\mathrm{mes}}-\varepsilon_{\mathrm{sol}}$ will increase in direct proportion to the square of the conductivity (Figure 4).

Thus, we come to the conclusion that the increase in the DC of aqueous solutions of electrolytes with an increase in their concentration, which was found experimentally in the first half of the last century, is not confirmed when the effect of electrical conductivity on the results of dielectric measurements is correctly taken into account. Therefore, for dilute solutions (*c* < 0.1 M), we can assume that their DC does not differ from the DC of pure water.

In connection with the development of measurement techniques at the end of the first half of the last century, it was possible to move to the region of centimeter wavelengths, and the experiments carried out showed that the DC of aqueous solutions of electrolytes decreases with increasing concentration [12]. These measurements were initiated in [33,34]. In these works, for the first time, a theoretical interpretation of the decrease in the DC with an increase in the electrolyte concentration was given. The authors [33,34] introduced the notion of dielectric decrement −dε_s_/dc (lowering the DC of water by one mole of electrolyte) and suggested that cations and anions have different effects on water. In this case, only cations are surrounded by dielectrically saturated water molecules. According to the dielectric saturation model [30], water molecules located near metal cations lose their orientational mobility [35]. In concentrated solutions, the formation of a cubic ionic lattice (body-centered for 1–1 and 2–2 electrolytes and face-centered for 1–2 electrolytes) is possible [36].

The results of dielectric measurements for dielectric properties of electrolyte and non-electrolyte solutions, carried out in the third quarter of the last century, are summarized and systematized in the reference book by Y.Y. Akhadov [12].

The dielectric decrement increases with increasing ion field strength. Therefore, at a given concentration, the greater the charge of the metal cation and the smaller its radius, the higher the decrease in the dielectric constant (dielectric decrement) will be. As a result, the following inequalities hold [12]:

ε(NaCl) > ε(MgCl_2_) > ε(AlCl_3_); ε(KCl) > ε(NaCl) > ε(LiCl) (13)

The dipole dielectric relaxation time τ of metal salts also decreases with an increase in the concentration of the electrolyte. In this case, for the values of τ, the same regularity of variation is observed as for ε_s_ (Equation (13)).

The determination of the static DC ε_s_ and the DR time τ is carried out on the basis of the experimentally measured active ε′ and reactive ε″ components of the complex DC ε*. In this case, to calculate ε_s_ and τ, the dipole component of the dielectric loss factor ε″_d_ is used. This value is obtained by subtracting from the measured value of ε″ the contribution of the ionic conductivity ε″_ion_ of the electrolyte solution [37]:

(14) $${\varepsilon \prime \prime }_{d}^{}=\varepsilon \prime \prime -{\varepsilon \prime \prime }_{\mathrm{ion}}^{}=\varepsilon \prime \prime -\frac{\kappa }{{\omega \varepsilon }_{0}}$$

With an increase in the electrolyte concentration, there is an increase in the specific EC κ of the solution. As a result, the values of ε″ and ε″_d_ approach each other, and the error in calculating the difference ε″−ε″_d_ increases. This error increases sharply with decreasing measurement frequency [37]. Table 2 shows the results of calculations of the values of ε′, ε″_d_, ε″_ion_, ε″ according to the Debye Equations (2) and (3) and the error of ε″_d_ for a 4 M NaCl solution [37].

Measurements of the dielectric characteristics of concentrated aqueous solutions of inorganic salts are carried out, as a rule, in the frequency range 1–50 GHz. The error in determining the dipole component of the dielectric loss factor δ(ε″_d_), required for calculating ε_s_ and τ, can reach several tens of percent (Table 2).

The ε″_d_ decreases and the ε″_ion_ increases with increasing temperature. This leads to an even greater increase in δ(ε″_d_) and an error in determining ε_s_ and τ. Therefore, to assess the dielectric characteristics of concentrated aqueous solutions of inorganic salts in a wide temperature range, it was proposed in [37] to use the independence of the ε_s_/ε_s_(H_2_O) and τ/τ(H_2_O) ratios on temperature. This makes it possible to estimate the values of ε_s_ and τ at high temperatures (up to *t* = 100 °C) on the basis of the ε_s_ and τ values measured with greater accuracy at low temperatures.

The time of the dipole dielectric relaxation τ of concentrated aqueous solutions of metal salts can also be estimated using two relaxation processes occurring in these solutions—dipole dielectric relaxation of solvent molecules with time τ(H_2_O) and ionic relaxation (relaxation of ionic atmospheres) with time θ [38]:

(15) $$\frac{1}{\tau_{\mathrm{solut}}}=\frac{1}{{\tau (H}_{2}O)}+\frac{1}{\theta }$$

The ionic relaxation time is calculated on the basis of the values of the static dielectric constant ε_s_ and the specific EC κ of solutions (θ = ε_s_ε_0_/κ). In dilute electrolyte solutions, θ << τ and the dipole relaxation time does not differ from the τ of water. In concentrated solutions, θ becomes comparable to τ of water (in a 4 M KCl solution, for example, θ ≈ 15 ps). As a result, the dielectric relaxation time of the solution τ_solut_, calculated using Equation (14), decreases with increasing concentration.

**Figure 5:**
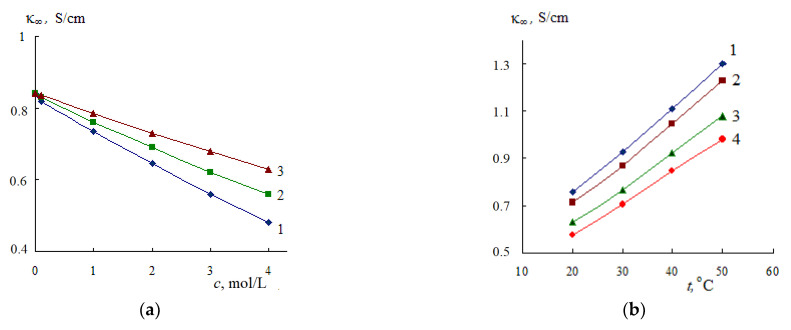
Dependences of the limiting HF EC of aqueous solutions LiCl (1), NaCl (2), KCl (3) from concentration, *t* = 25 °C (**a**) and NaCl solutions from temperature, concentration: 1—0(H_2_O), 2—1, 3—3, 4—5 mol/L (**b**).

The limiting HF EC of aqueous electrolyte solutions calculated on the basis of ε_s_ and τ [12] according to Equation (1) also decreases with increasing solution concentration (Figure 5a).

An increase in temperature leads to an increase in the limiting HF EC of the solution; however, in the entire investigated concentration range, κ_∞_ of the solution does not exceed the value of κ_∞_ of water (Figure 5b).

Concluding the consideration of the dielectric characteristics of aqueous solutions of electrolytes, it should be noted that in solutions of some electrolytes, for example BeSO_4_ and CuSO_4_, an increase in static DC was found with increasing concentration, which is explained by the contribution of dipole relaxation of ion pairs formed in the solution [39,40]. In [39], for example, the static permittivity of a 1 M BeSO_4_ solution increases in comparison with pure water based on the results of measurements of ε′ and ε″ in the frequency range 0.1–2.5 GHz. At such low frequencies, as follows from Table 2, a large error in determining the dipole component ε″, and hence the ε_s_ value of the solution, is possible.

## 4. Dielectric Characteristics of Aqueous Non-Electrolyte Solutions

**Figure 6:**
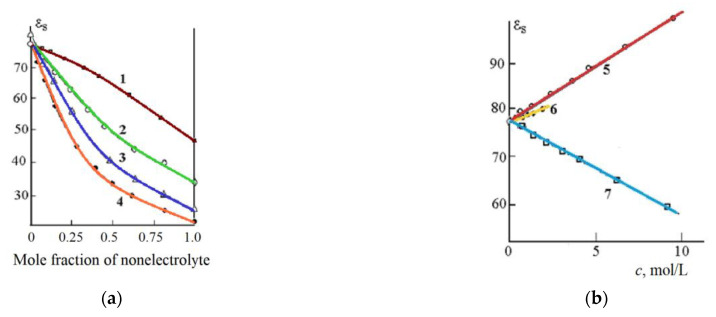
Dependence of the static dielectric constant of mixtures of water with DMSO (1), methanol (2), ethanol (3), acetone (4) (**a**); urea (5), thiourea (6) and formaldehyde (7) (**b**) from the concentration of the non-aqueous component; *t* = 25 °C [51].

When considering the dielectric characteristics of some non-electrolyte solutions, the data of the reference book [12] and the results of dielectric studies of aqueous-organic mixtures published in [41,42,43,44,45,46,47,48,49,50] were used. The static dielectric constant decreases with increasing concentration of most non-electrolytes (Figure 6). This decrease is caused by the destruction of water hydrogen bonds by non-electrolyte molecules.

The addition of urea and thiourea to water leads to an increase in the static DC of the solution (curves 5 and 6, Figure 6b). This increase is apparently caused by the formation of additional hydrogen bonds between water molecules and non-electrolyte (urea and thiourea).

**Figure 7:**
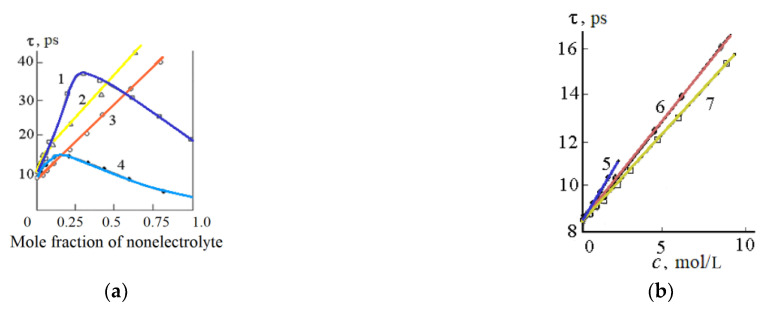
Dependence of the dielectric relaxation time of mixtures of water with DMSO (1), ethanol (2), methanol (3), acetone (4) (**a**); thiourea (5), urea (6) and formaldehyde (7) (**b**) from the concentration of the non-aqueous component; *t* = 25 °C [51].

The time of dipole dielectric relaxation also changes in different ways with an increase in the concentration of the nonelectrolyte (Figure 7).

In mixtures of water with methanol, ethanol, urea, thiourea, ammonia, and formaldehyde, the relaxation time increases with an increase in the non-electrolyte concentration. In mixtures of water–acetone and water–DMSO, a maximum is observed on the curves “τ—composition” (Figure 7b). The extreme character of the “τ—composition” curves in aqueous-organic mixtures can be associated with the formation of compounds between the components of the solution. For example, in mixtures of water with acetone and DMSO, the formation of compounds of the composition (CH_3_)_2_CO·4H_2_O and (CH_3_)_2_SO·2H_2_O is possible. Another option for explaining the presence of a maximum can be the effect of stabilization of the water structure by non-electrolyte molecules. This effect takes place in the range of concentrations of the non-aqueous component 15 < *X* < 30 mol.%, which coincides with the maximum observed in Figure 7a. The maximum on the “τ—composition” curves is also observed for aqueous solutions of anetonitrile [41] and dimethylformamide [49,50].

**Figure 8:**
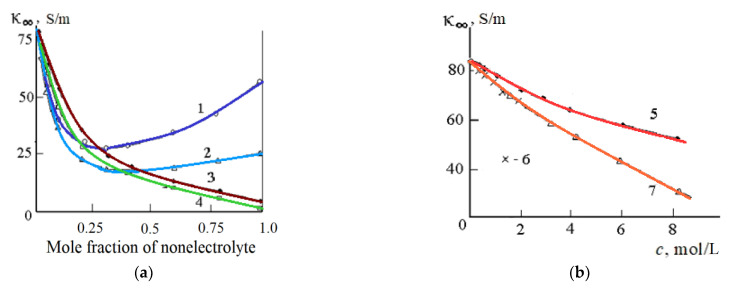
Dependence of the limiting HF EC of mixed solvents from the concentration (**a**): acetone (1), DMSO (2), methanol (3), ethanol (4); (**b**): urea (5), thiourea (6), and formaldehyde (7); *t* = 25 °C [51].

Despite the different nature of the change with an increase in the concentration of the non-aqueous component of the dielectric characteristics ε_s_ (Figure 6) and τ (Figure 7) of aqueous-organic mixtures, the limiting high-frequency EC of these mixtures decreases when the non-aqueous component is added to the water (Figure 8).

The most significant decrease in the limiting HF EC (κ_∞_) of aqueous-organic mixtures is observed at relatively low (*X* < 20 mol.%) concentrations of nonelectrolyte (Figure 8a). In some cases (in mixtures of water with acetone and DMSO) at *X* = 30–40 mol.%, a minimum appears on the “κ_∞_—composition” dependence (Figure 8a). The minimum on the “κ_∞_—composition” dependence is also observed in aqueous solutions of acetonitrile and dimethyl sulfoxide.

Concluding the consideration of the dielectric characteristics of aqueous solutions of non-electrolytes, it should be noted that to estimate the values of ε_s_, τ, and κ_∞_, as in the case of electrolyte solutions, one can use the independence of the ε_s_/ε_s_(H_2_O), τ/τ(H_2_O), and κ/κ_∞_(H_2_O) ratios on temperature. For the solutions of non-electrolytes considered above, this independence is fulfilled in the temperature range of 10–40 °C [51].

## 5. Absorption of Microwave Energy by Polar Solvents and Their Solutions

Microwave chemistry is currently being intensively developed in a theoretical direction and is finding practical applications [52]. Exposure to microwave irradiation can significantly increase the rate of chemical reactions [53]. An important role is played by the intensification of chemical processes occurring in aqueous solutions under the conditions of microwave irradiation [54].

The most important advantage of using microwave irradiation in chemistry and chemical technology is that the energy of this radiation is absorbed throughout the entire volume of the reaction mixture. In this case, by changing the parameters of microwave radiation, one can selectively act on any components of the reaction mixture. Despite the possibility of widespread use of microwave irradiation in chemistry, its use is hampered by the lack of theoretical concepts describing the absorption of a high-frequency electromagnetic field by a substance. This primarily concerns solutions of electrolytes and non-electrolytes in polar solvents, in which a significant number of chemical and technological processes take place.

When a high-frequency electromagnetic field is imposed, the intensity *E* of the specific active power *P* absorbed by the substance is directly proportional to the value of its active component of high-frequency electrical conductivity κ′:

*P* = κ′*E*^2^ = ωε_0_ε″ (16)

where *E* is the electromagnetic field strength.

In the practice of microwave chemistry, when carrying out processes in polar solvents, the frequency corresponding to the maximum dielectric loss factor ε″ is usually chosen. However, the maximum active conductivity κ′ and therefore the maximum absorbed power *P* do not correspond to the maximum dielectric loss factor ε″. As shown above, with an increase in the frequency of the electromagnetic field, an increase in the active component HF EC κ′ occurs, and under the condition ωτ >> 1, κ′ reaches its limiting maximum value equal to κ_∞_. The dielectric loss factor ε″ reaches its maximum value under the condition ωτ = 1. In this case, Equation (5) is simplified:

κ′ = κ_∞_/2 (17)

Thus, at the maximum of the dielectric loss factor ε″, the active conductivity is only half of the value of the limiting HF EC of the solvent κ_∞_, and therefore the power released in the substance will be half of the maximum possible. Hence, the optimal condition for carrying out processes in polar solvents under microwave irradiation conditions is the choice of frequency corresponding to the condition ωτ >> 1. In this case, the power *P* absorbed by the reaction mixture will be maximum.

Since a significant number of processes in solutions are carried out at a frequency of 2455 MHz, in this work, a theoretical and experimental study of the absorption of microwave energy by polar solvents and their solutions at this frequency was carried out. Microwave heating of polar solvents and solutions at 2455 MHz was studied using the Discover Bench Mate microwave system.

HF heating was carried out in a special test tube with a magnetic stirrer. The volume of the test solution was 5.0 mL, and the power of the microwave system was 10 W. Temperature readings were recorded every 10 s. The HF heating time was 80 s. The liquid temperature was monitored with a non-contact infrared sensor. To determine the rate of HF heating *V* of the polar solvents under study, the graphs of temperature *t* versus time τ were analyzed:

*t* = *t*_o_ + *V*τ, (18)

from which the HF heating rate *V* was determined. To check the reproducibility of the *t = f*(τ) curves, the experiment for each solvent was repeated at least three times. The relative error in determining the rate of HF heating *V* did not exceed 3%.

**Figure 9:**
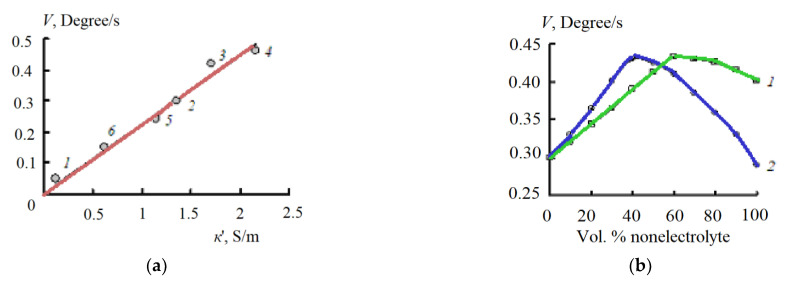
Dependence of the HF heating rate *V* of acetone (1), water (2), DMSO (3), methanol (4), ethanol (5), and propanol (6) from the HF EC κ′ of a polar solvent (**a**) and aqueous solutions of DMSO (1) and DMF (2) from the concentration of nonelectrolyte (**b**).

As a result of the studies carried out, it was found that the maximum rate of HF heating occurs in the case of DMSO and methanol. Despite the relatively high value κ_∞_ of acetone (Table 1), the rate of its high-frequency heating was the lowest. To explain the results obtained by Equation (5), the HF EC of the studied polar solvents was calculated and the dependence of the HF heating rate on HF EC was plotted (Figure 9a).

As can be seen from Figure 9a, the HF heating rate of a polar solvent *V* increases in direct proportion to its high-frequency electrical conductivity κ′ at a frequency of 2455 MHz. This fact is consistent with the dependence of the absorbed power on the HF EC of a polar solvent, described by Equation (16).

In all aqueous solutions of nonelectrolytes studied in this work, with an increase in the content of nonelectrolyte, the rate of HF heating passes through a maximum [55,56]. At the same time, the maximum on the *V*-composition curves is observed not only in mixtures of water with dimethylformamide and dimethyl sulfoxide (Figure 9b), which are characterized by the presence of a maximum on the τ-composition curves (Figure 7a); the maximum on the *V*-composition curves also occurs in the case of aqueous solutions of methanol, ethanol, and propanol. For these solutions at temperatures of 10, 25, and 40 °C, according to Equation (5), the values of high-frequency conductivity at a frequency of 2455 MHz were calculated. The results of these calculations are presented in Table 3.

As follows from the data of these tables, for all solutions with an increase in alcohol concentration, the value of κ′ passes through a maximum. The presence of this maximum explains the existence of a maximum in the dependences of the rate of high-frequency heating of aqueous solutions of alcohols from the concentration of nonelectrolyte.

With an increase in the alcohol content, the limiting HF EC κ_∞_ of all aqueous-organic solutions decreases (Figure 8). At the same time, the active HF EC calculated at a frequency of 2455 MHz with an increase in the content of the organic component passes through a maximum. The position of this maximum coincides with the extremum on the dependences of the HF heating rate of solutions on their composition (Figure 9b, Table 3). However, the different nature of the maximum should be noted. In mixtures of water with acetone, acetonitrile, DMSO, and DMF, the HF EC extremum is explained by the existence of a maximum in the dependence of the dielectric relaxation time on the composition of the aqueous-organic mixture [12,41,42,43,44,45,46,47,48,49,56]. In aqueous solutions of alcohols, as noted above, a monotonic increase in the dipole relaxation time with an increase in the alcohol content is observed [12,57,58].

According to Equation (5), the active component of the high-frequency conductivity κ′ depends in a complex way on the frequency of the electromagnetic field ω, the static dielectric constant ε_s_, and the time of dipole dielectric relaxation τ. If the condition (ωτ)^2^ << 1 is satisfied, then Equation (5) is transformed to the form:

κ′ = κ_∞_(ωτ)^2^ = ε_s_ε_0_ω^2^τ (19)

In the considered aqueous-alcoholic solutions, when passing from water to alcohol, the static DC decreases by 3–4 times [12,57,58], and the time of dipole dielectric relaxation increases by 10–30 times [12,57,58]. Therefore, under the condition (ωτ)^2^ << 1, the HF EC κ′ will increase with an increase in the alcohol content in the solution according to Equation (19).

Under the condition (ωτ)^2^ >> 1, Equation (5) turns into Equation (1) (κ′ = κ_∞_). As a result, according to Equation (1), the HF EC will decrease with an increase in the alcohol concentration since an increase in the concentration of alcohol leads to a decrease in the static DC and an increase in the dielectric relaxation time.

At a frequency of 2455 MHz, the condition (ωτ)^2^ = 1 is satisfied if the dielectric relaxation time is 64.8 ps. Thus, in the solutions under study, with a change in the concentration of alcohol and temperature, both the condition (ωτ)^2^ << 1 (for water at 40 °C (ωτ)^2^ = 0.0083) and the condition (ωτ)^2^ >> 1 (for propanol at 10 °C (ωτ)^2^ = 26.3). As a result, with an increase in alcohol concentration, the HF EC κ′ at a frequency of 2455 MHz passes through a maximum.

Since for all investigated aqueous-alcoholic solutions the limiting HF EC κ_∞_ increases with increasing temperature and with decreasing alcohol content, under the condition (ωτ)^2^ >> 1, the effect of HF irradiation on mixtures with a low alcohol content in the region of elevated temperatures will be the most effective.

A different picture is observed under microwave irradiation of the investigated aqueous-alcoholic solutions at a frequency of 2455 MHz. With an increase in the alcohol content, the HF conductivity κ′ passes through a maximum (Table 3). Consequently, the effect of HF irradiation on solutions containing ~40–60 vol.% methanol, ~30–50 vol.% ethanol, and ~25–40 vol.% propanol will be most effective. An increase in temperature leads to a decrease in the HF EC κ′ in aqueous solutions of methanol, ethanol, and propanol (Table 3). Therefore, the effect of an HF field with a frequency of 2455 MHz on the processes taking place in the considered mixed solvents at elevated temperatures will be less effective.

The absorption of microwave energy by aqueous solutions of electrolytes is considered using the example of an aqueous solution of NaCl. Since, according to Equation (16), the power of microwave radiation absorbed by the solution is proportional to its HF EC, in [37] the values of the limiting HF EC κ_∞_ and HF EC κ′ at the frequency 2455 MHz were calculated for an aqueous solution of NaCl in the concentration range 0–5 M at temperatures 20, 30, 40, and 50 °C (Table 4).

An increase in the concentration of NaCl solution leads to a decrease in both the limiting HF EC κ_∞_ and HF EC κ′ at the frequency 2455 MHz. This decrease is due to a more significant decrease in the static DC in going from water to a 5 M NaCl solution than the decrease in the relaxation time [37]. An increase of temperature has a different effect on the values of κ_∞_, and κ′. The limiting HF EC κ_∞_ increases, and the HF EC at the frequency 2455 MHz κ′ decreases with increasing temperature (Table 4). The increase of the limiting HF EC κ_∞_ with increasing temperature is due to the fact that when heated from 10 to 50 °C, the static DP decreases by only 1.24 times, while the relaxation time is 2.75 times [37].

The decrease in the HF EC κ′ at the frequency 2455 MHz is caused by a significant decrease of the product ωτ of the solution with increasing temperature. At a temperature of 10 °C, for example, the product ωτ for a 2 M NaCl solution is 0.174, and at 50 °C, it is 0.0632. Since the static DC and dielectric relaxation time decrease with increasing temperature and concentration, the HF EC κ′ of NaCl solutions calculated at a frequency of 2455 MHz according to Equation (19) also decreases (Table 4). Thus, according to the theoretical analysis, the absorption of HF electromagnetic energy by an NaCl solution at frequency 2455 MHz should decrease with increasing electrolyte concentration.

To check the obtained regularities at the frequency 2455 MHz, studies of high-frequency heating of sodium chloride solutions were carried out. As a result of the studies carried out, it was found that the HF heating rate decreases from 0.30 (water) to 0.26 deg/s (5 M NaCl). The resulting decrease in the rate of HF heating is thus due to a decrease in the HF EC of the NaCl solution κ′ with increasing concentration (Table 4), since the value of the absorbed energy of the HF field is proportional to the HF conductivity (Equation (16)). On the other hand, the decrease in the rate of high-frequency heating of NaCl solutions with increasing salt content can be associated with the processes of ion solvation. Water molecules in the solvation shells of ions lose their orientational mobility due to the effect of dielectric saturation. The effect of dielectric saturation causes a decrease in the HF EC of electrolyte solutions with an increase in their concentration. As a result, the water molecules in the solvation shells of ions reduce their ability to reorient in the external electromagnetic field and, consequently, to absorb the HF energy of the electromagnetic field. Therefore, we come to the conclusion that the effect of the HF field with a frequency of 2455 MHz on the processes occurring in aqueous solutions of electrolytes, as in the case of aqueous solutions of non-electrolytes, at elevated temperatures will be less effective.

## 6. Specific Electrical Conductivity of Electrolyte Solutions and the Limiting High-Frequency Conductivity of a Polar Solvents

**Figure 10:**
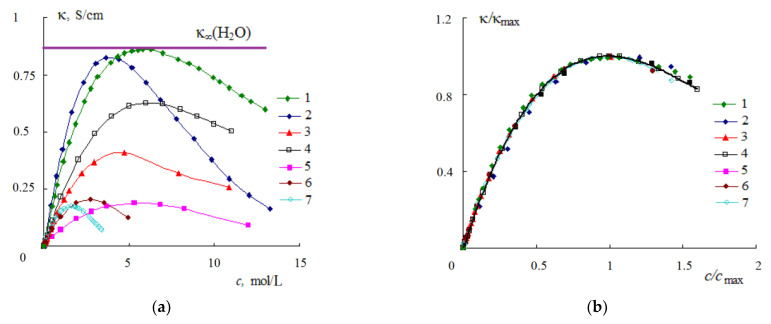
Dependence of the specific EC of aqueous solutions HNO_3_ (1), H_2_SO_4_ (2), NaOH (3), KOH (4), LiCl (5), CaCl_2_ (6), and LaCl_3_ (7) on concentration according to [59] (**a**) and the reduced EC κ/κ_max_ of these solutions versus the reduced concentration *c*/*c*_max_ (**b**); *t* = 25 °C.

With sufficient solubility, an increase in concentration *c* leads to an increase in the specific electrical conductivity κ of solutions as a result of an increase in the number of ions in dilute solutions. However, in concentrated solutions, a maximum appears on the κ − *c* dependence as a result of the association of ions, which occurs due to the fact that there are not enough solvent molecules for their solvation (Figure 10).

For aqueous solutions of metal salts, the maximum is observed at a concentration corresponding to the full solvation border (FSB). According to the data in Figure 10, FSB occurs at a concentration of ~5 M for a LiCl solution, ~2.8 M for a CaCl_2_ solution, and ~1.6 M for a LaCl_3_ solution. A comparison of the calculated values of the concentrations of solutions of lithium, magnesium, calcium, and lanthanum chlorides corresponding to FSB with the concentrations of the maximum electrical conductivity *c*_max_ [59] is given in Table 5.

The concentration corresponding to the FSB was calculated by dividing the concentration of water in a solution *c*(H_2_O) by the number of water molecules bound to ions *N*(H_2_O). The concentration of water molecules *c*(H_2_O) was determined based on the values of the densities of solutions ρ given in the reference book [59]. The number of water molecules bound to ions *N*(H_2_O) at the FSB concentration was determined by summing the coordination numbers of the ions. In this case, the following values of the coordination numbers of ions were taken: lithium—4; magnesium, calcium and chlorine—6; cation and anion in a solution of lanthanum chloride—8. All the above values of ρ, *c*(H_2_O), *N*(H_2_O), *c*(FSB), as well as the maximum value of the specific EC κ_max_ of solutions are given in Table 5.

For all solutions except LiCl, the difference between the calculated values of *c*(FSB) and the concentrations corresponding to the maximum specific EC *c*_max_ does not exceed 3%. The difference of 6.4% for the LiCl solution is apparently due to the absence of the concentration values given in the reference book [59] and the corresponding values of the specific EC near κ_max_ (reference [59] gives the values of κ at concentrations 3.70, 5.33, and 6.85 M). Thus, the coincidence of the experimental values of *c*_max_ and the calculated values of *c*(FSB) indicates that the maximum in the dependence of the specific EC of the solutions under consideration is observed at a concentration corresponding to the limit of complete solvation.

The maximum specific conductivity occurs in aqueous solutions of strong inorganic acids. The κ_max_ values of these acids are close, practically independent of their nature, and amount to 0.84 ± 0.02 S/cm at a temperature of 25 °C [59]. This value coincides with the value of the limiting high-frequency conductivity κ_∞_ of water (Table 1). We believe that the value of κ_∞_ water, as it were, limits the maximum specific conductivity κ_max_ of aqueous electrolyte solutions. For aqueous solutions of strong inorganic acids κ_max_ = κ_∞_(H_2_O), and for solutions of metal salts, it is a certain part of it, proportional to the salt concentration *c* and the number of water molecules associated with one mole of electrolyte *N* [7]:

(20) $$\kappa =\frac{cN}{c{(H}_{2}O)}\times \frac{\varepsilon_{0}\varepsilon_{s}}{\tau }=K\times \kappa_{\infty }$$

In Equation (20), *c*(H_2_O) is the concentration of the solvent (for dilute aqueous solutions ***c***(H_2_O) = 55.5 mol/L), and *K* is the fraction of water molecules associated with electrolyte ions.

Using Equation (20), it is possible to estimate the value of the specific EC of concentrated aqueous solutions of alkali metal halides, using the coordination number of the metal cation as the value of *N*. For 1 M aqueous solution of NaCl, for example, the EC value calculated by Equation (20) is 8.48 S/m [38] and, with an error that does not exceed 1.5%, coincides with the experimental value of κ of this solution (8.61 S/m [59]).

The dependences κ−*c* shown in Figure 10a can be generalized (reduced to a single dependence) if the reduced electrical conductivity (κ/κ_max_) and the reduced concentration *c*/*c*_max_ are used. In this case, the experimental values of the specific electrical conductivity of aqueous solutions of acids, alkalis, and metal salts fit into a single curve (Figure 10b). A single dependence in the coordinates (κ/κ_max_) = *f*(*c*/*c*_max_) takes place in a wide temperature range for both strong [60,61] and weak [62,63] electrolytes.

**Figure 11:**
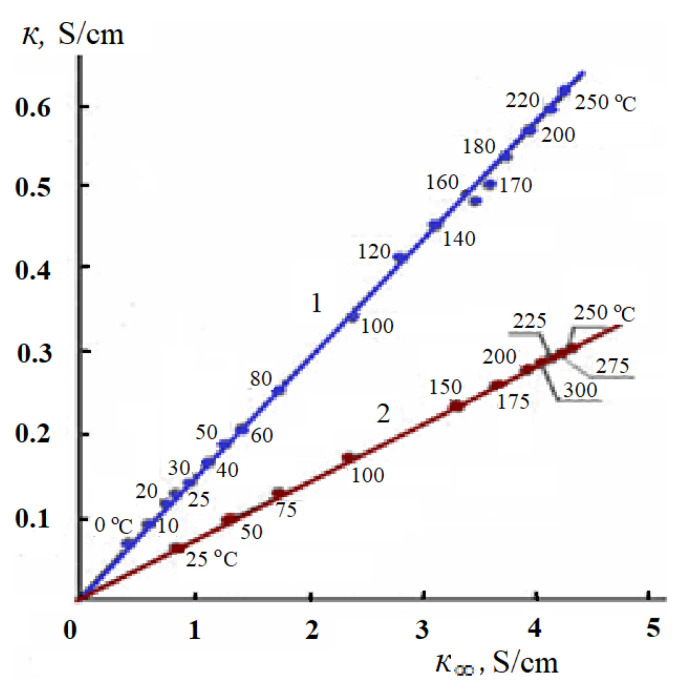
Dependence of the specific electrical conductivity of 0.1 (1) and 0.05 M (2) KCl solutions on the limiting HF EC of water; temperature values are shown in the graph; at temperatures above the boiling point of the solutions, results are given along the liquid–vapor coexistence curve.

According to Equation (20), the specific EC of the electrolyte solution κ should increase in direct proportion to the value of the limiting HF EC of the solvent κ_∞_ with increasing temperature. The study of the temperature dependences of the specific EC of aqueous solutions of inorganic salts shows that the proportionality described by Equation (20) is fulfilled in a wide temperature range: with increasing temperature, the specific EC of these solutions increases in direct proportion to the limiting HF EC of water [38]. It is interesting to note that the considered proportionality is fulfilled not only in the region where the specific EC of the solution increases with increasing temperature (0–250 °C), but also at temperatures exceeding 250 °C, where there is a decrease in the specific EC of metal salt solutions and the limiting HF EC of water with increasing temperature [38]. Figure 11 shows, as an example, the dependences κ−κ_∞_ for 0.1 and 0.05 M KCl solutions, which confirm the regularity described by Equation (20).

Consequently, the dielectric characteristics of water, and in particular, its static dielectric constant ε_s_ and the dielectric relaxation time τ determine the conductivity of aqueous solutions of electrolytes. The temperature dependence of the specific EC is determined by the change with temperature of the static dielectric constant and the time of the dipole dielectric relaxation (Equation (20)).

Equation (20) describes the relationship between the specific electrical conductivity κ of solutions and the dielectric properties of the solvent ε_s_ and τ. Passing from specific κ to equivalent λ (λ = κ/*c*) electrical conductivity, we obtain:

(21) $$\lambda =\frac{\kappa }{c}=\frac{N}{c{(H}_{2}O)}\times \frac{\varepsilon_{0}\varepsilon_{s}}{\tau }=K^{\prime }\times \kappa_{\infty }$$

The λ value approaches the equivalent EC at infinite dilution λ_0_, and κ_∞_ approaches the κ_∞_(H_2_O) value with a decrease of the solution concentration. As a result, we obtain an equation relating the electrical conductivity at infinite dilution λ_0_ with the dielectric properties of water:

(22) $$\frac{\lambda_{0}\tau }{\varepsilon_{0}\varepsilon_{s}}=\frac{\lambda_{0}}{\kappa_{\infty }{(H}_{2}O)}=const,$$

In contrast to Walden’s rule (λ_0_η = *const* [1]), the ratio λ_0_/κ_∞_(H_2_O) remains constant in a wide temperature range not only for aqueous, but also for non-aqueous solutions of inorganic salts [64]. Therefore, Equation (22) can be recommended for describing the temperature dependence of the equivalent electrical conductivity at infinite dilution of inorganic salt solutions.

Since the value of the dipole relaxation time is proportional to the ratio η/*T*, Equation (22) differs from Walden’s rule in that it does not contain the product ε_s_*T* in the denominator. As shown in [64], for aqueous solutions of inorganic salts in the temperature range 0–100 °C, the λ_0_η/(ε_s_*T*) ratio remains unchanged; therefore, the Walden rule is recommended to be used in the form:

(23) $$\frac{\lambda_{0}\eta }{\varepsilon_{s}T}=const.$$

In order to check the regularities shown in Figure 10 and Figure 11 in non-aqueous solutions, in this work, in a wide range of concentrations, we measured the conductivity of some ionic liquids in acetonitrile (AN), dimethylformamide (DMF), and dimethyl sulfoxide (DMSO). To remove moisture, the ionic liquids were kept at a temperature of 60 °C under vacuum for three hours. The electrical conductivity of the solutions was measured using an E 7–20 digital automatic AC bridge in the frequency range 0.5–50 kHz. In order to exclude the influence of polarization processes on the results of conductometric measurements, the sought resistance of solutions was found by extrapolating its measured value *R* to an infinite frequency in the coordinates *R*−1/F [32]. The constant of the conductometric cell was determined using KCl solutions [65] with a concentration of 0.01, 0.1, and 1.0 mol/kg, the electrical conductivity of which was measured with high accuracy [66]. The accuracy of thermostating solutions was ±0.02 °C. The error in measuring the specific EC of solutions did not exceed 0.5%.

**Figure 12:**
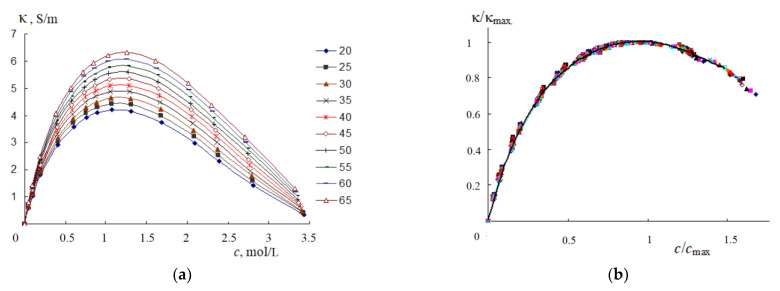
Dependence of the specific EC of solutions ([bmim][NTf2]) in acetonitrile on concentration (**a**); temperatures (°C) shown in the graph and reduced EC versus normalized concentration for four ionic liquids (**b**).

Figure 12a shows the concentration dependence of the specific EC of 1-butyl-3-methylimidazolium bis {(trifluoromethyl) sulfonyl} imide ([bmim][NTf2]) solutions in acetonitrile. Similar dependences were obtained for solutions of 1-butyl-3-methylimidazolium trifluoromethane sulfonate, 1-octyl-3-methylimidazolium sulfonate, and 1-butyl-3-methylpyridinium bis {(trifluoromethyl) sulfonyl} imide in acetonitrile. The values of the reduced EC κ/κ_max_ (more than four hundred values) of all four ionic liquids fit well into one curve in the coordinates (κ/κ_max_) − *f*(*c*/*c*_max_), as seen in Figure 12b.

The reduced EC values also fit well into a single curve in coordinates (κ/κ_max_) − *f*(*c*/*c*_max_) for solutions of trihexyl(tetradecyl) phosphonium chloride ([P66614]Cl) in acetonitrile (AN), 1-butyl-3-methylpyridinium bis {(trifluoromethyl) sulfonyl} imide and 1-butyl-3-methylimidazolium bis {(trifluoromethyl) sulfonyl} imide dimethylformamide, and 1-butyl-3-methylpyridinium bis {(trifluoromethyl) sulfonyl} imide and ([P66614]Cl in dimethyl sulfoxide. Thus, the generalized regularity established earlier (Figure 10b) is confirmed for solutions of all ionic liquids in acetonitrile, dimethylformamide, and dimethyl sulfoxide.

**Figure 13:**
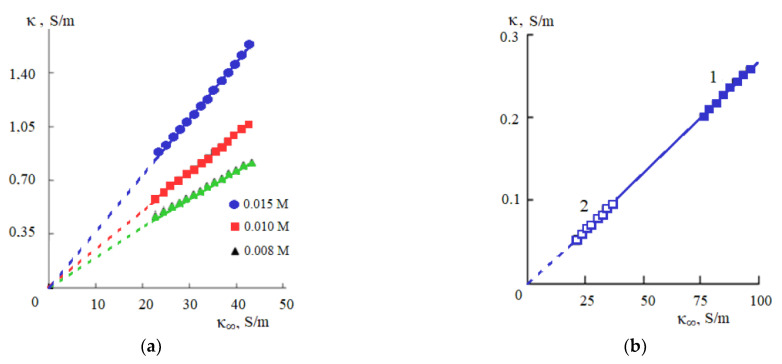
Dependences κ−κ_∞_ for solutions of [bmim][NTf_2_] in dimethylformamide (**a**) and 0.02 M solution of [P66614]Cl in acetonitrile (1) and dimethylsulfoxide (2) (**b**).

For all investigated ionic liquids in acetonitrile, dimethylformamide, and dimethylsulfoxide, another regularity is fulfilled: just as in aqueous solutions, the specific EC of ionic liquids increases with increasing temperature in direct proportion to the limiting HF EC of the organic solvent. Figure 13 shows as an example the dependences κ–κ_∞_ for solutions of [bmim][NTf_2_] in dimethylformamide (Figure 13a) and 0.02 M solution of [P66614]Cl in acetonitrile and dimethylsulfoxide (Figure 13b).

Specific conductivity passes through a maximum with an increase in the concentration of electrolyte in aqueous solutions (Figure 10a) and in non-aqueous solutions of ionic liquids (Figure 12a). To clarify the regularity of the appearance of the maximum on the κ−*c* curves, let us consider how the distance between ions in solutions changes.

## 7. Distance between Particles (Ions and Molecules) in Solutions

The properties of electrolyte solutions, in particular their electrical conductivity, depend not only on the dielectric properties of the solvent. The interaction between ions, their solvation and association, are determined by the distance, which decreases with increasing concentration. A possible variant of estimating the distance d between particles in solutions is considered below. In this case, the distance *d* is successively evaluated in an ideal gas, in a liquid, in a non-electrolyte solution, and in an electrolyte solution. We begin to analyze the distance between particles with an ideal gas, in which the absence of intermolecular interaction is assumed.

### 7.1. Distance between Ideal Gas Molecules under Normal Conditions

**Figure 14:**
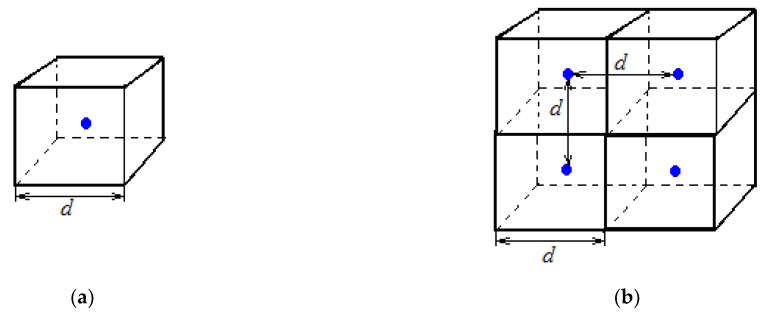
Ideal gas molecule location at the center of a cube with a volume of 37.2∙10^−27^ m^3^ under normal conditions (**a**) and arrangement of cubes with ideal gas molecules (**b**).

Consider an ideal gas under normal conditions and occupying a volume of *V*_0_ = 22.4∙10^−3^ m^3^. We will assume that each ideal gas molecule is located in the center of the cube (Figure 14a), the volume of which *V*_1_ is equal to *V*_1_ *=* V_0_*/N*_A_ = 22.4∙10^−3^/(6.022∙10^23^) = 37.2∙10^−27^ m^3^. In this case, the value *V*_1_*N*_A_ *= V*_0_ = 22.4∙10^−3^ m^3^.

The length of the edge *d* of the cube under consideration (Figure 14) will be:

(24) $$d=\sqrt[{3}]{V_{1}}=33.4\times 10^{-10}m=33.4Å$$

Thus, we obtain the average distance between ideal gas molecules under normal conditions, equal to 33.4∙10^−10^ m (33.4 Å). This value is the same for all gases under normal conditions.

In the case of water vapor, under normal conditions, the distance between the centers of two neighboring molecules will also be 33.4 Å. The radius of a water molecule is considered to be *r* = 1.38∙10^−10^ m (1.38 Å). The diameter of a water molecule is *d* = 2.76 Å. Then, at a distance of 33.4 Å between the centers of neighboring molecules H_2_O, 12 water molecules of water can be located. It can be assumed that in this case, there is practically no intermolecular interaction between water molecules, and its properties can be described by the equations used to describe the properties of ideal gases.

### 7.2. Distance between Molecules in an Aqueous Solution of Non-Electrolytees

The calculation between the dissolved substance molecules in the solution is possible only under the condition that the concentration *c* of the solution is expressed in mol/L, that is, in units of molyarity (M). By accepting a solution equal to one liter and denoting its molar concentration with *c*, we get:

(25) $$d=\sqrt[{3}]{\frac{10^{-3}}{N_{A}c}=}\frac{11.84\times 10^{-10}}{\sqrt[{3}]{c}}m=\frac{11.84}{\sqrt[{3}]{c}}Å$$

From the resulting expression (25), it follows that in 1 M aqueous solution of non-electrolyte, the distance between its molecules is 11.84 Å. Not more than four water molecules may be placed between the non-electrolithic molecules in this solution (11.84/2.76 = 4.29), and taking into account the size of the non-electrolyte molecule, no more than three.

### 7.3. Distance between Ions in Electrolyte Solutions

In an aqueous electrolyte solution, the amount of dissolved particles depends on the degree of dissociation. In dilute electrolyte solutions, the dissociation degree is 100 % and this amount is equal to *nc*, where *n* is the number of particles, which dissociates one mol of the dissolved substance, and *c* is a molar concentration. As a result, we obtain, to calculate the distance between the particles of the dissolved substance in the solution *d,* the following expression:

(26) $$d=\sqrt[{3}]{\frac{10^{-3}}{nN_{A}c}=}\frac{11.84\times 10^{-10}}{\sqrt[{3}]{nc}}m=\frac{11.84}{\sqrt[{3}]{nc}}Å.$$

For non-electrolyte *n* = 1; for I–I and II:II electrolyte (NaCl, MgSO_4_) *n* = 2; for I–II and II:I (CaCl_2_, Na_2_SO_4_)—*n* = 3; for I–III and III:I (AlCl_3_, Na_3_PO_4_)—*n* = 4; for II-III (Al_2_(SO_4_)_3_)—*n* = 5. Using the obtained Equation (26), the distance *d* between molecules (*n* = 1) and ions (*n* > 1) in solutions for various concentrations *c* and electrolyte types *n* are assumed under the assumption that the degree of dissociation of electrolyte is 100%. The results of these calculations are shown in Table 6.

It is important to note that the distances given in Table 6 for various concentrations are valid for both aqueous and non-aqueous solutions. Based on the values given in the tables, the following conclusions can be drawn.

In dilute aqueous solutions (at *c* ≤ 0.01 M), the distance between ions in the solution is greater than in gaseous water under normal conditions (33.4 Å). In these solutions, 12 or more water molecules can be placed between ions. To describe the properties of aqueous solutions at *c* < 0.01 M, the the classical electrolyte theory (Debye–Hückel) can be used. The size of the molecules of non-aqueous solvents is usually larger than the size of the water molecule. Therefore, the concentration of a non-aqueous solution in which 12 solvent molecules can be located between the ions will be less than 0.01 M. Therefore, the Debye–Hückel theory is valid for non-aqueous solutions, the concentrations of which are significantly less than 0.01 M.

In concentrated solutions (at *c* ≥ 1.0 M), the distance between the ions is such that 12 water molecules cannot be located between them. Therefore, to describe these solutions, it is necessary to take into account the interaction between ions. As an example, let us estimate the number of water molecules that can be located between ions in a 5 M LiCl solution on the assumption of complete dissociation of the electrolyte. The distance between the centers of ions in this solution is 5.50 Å. Taking into account the radii of the Li^+^ ion (~0.7 Å) and the Cl^−^ ion (~1.8 Å), we subtract the sum of the ion radii (0.7 + 1.8) = 2.5 Å from 5.50 Å, and we obtain that for molecules of water in this solution, the value remains 3.0 Å. This value is less than the diameter of two water molecules (5.52 Å). Thus, all ions (cation and anion) in a 5 M solution of lithium chloride cannot be solvated, and the association of ions in this solution occurs with the formation of ion pairs separated by one molecule of solvent.

From Equation (26), it is possible to calculate the concentration at which a certain number of solvent molecules are located between the ions. We transform Equation (26) and obtain the following expression for the concentration:

(27) $$c=\frac{10^{-3}}{d^{3}nN_{A}}=\frac{1.66\times 10^{3}}{d^{3}n},\mathrm{mol}/L$$

In Equation (27), the value of *d* is equal to the sum of the radii of the cation and anion, and the diameters of water molecules. For an aqueous solution of LiCl, *d* is 5.26 Å (0.7 + 1.8 + 2.76). Substituting the value *d* = 5.26 Å into Equation (26), we obtain the concentration of the LiCl solution, which is 5.70 mol/L. This value is close to the concentration corresponding to the maximum in the dependence of the specific EC of the LiCl solution (5.33 mol/L), as seen in Table 5.

In non-aqueous solutions of ionic liquids, the maximum specific EC occurs in the concentration range of 1.0–1.5 mol/L. (Figure 12a). At these concentrations, the distance between the centers of the ions, calculated by Equation (26), turns out to be 11.84–10.34 Å. Preliminary estimates show that at such values of *d*, solvent molecules cannot be located between the ions of ionic liquids. Therefore, contact ion pairs may form in non-aqueous solutions of ionic liquids. It is their formation that leads to a decrease in the specific EC at *c* > *c*_max_ (Figure 12a). Further studies will provide additional information on the processes of ion solvation in polar solvents.

## 8. Conclusions

In the field of frequency dispersion of the dielectric permeability, polar solvents pass from the class of dielectrics to the class of conductors. Their high-frequency conductivity increases with increasing frequency and, under the condition ωτ >> 1 (ω = 2π*F*, τ is the dipole dielectric relaxation time), reaches its limiting value κ_∞_. The limiting high-frequency electrical conductivity κ_∞_ is determined by the ratio of the static dielectric constant ε_s_ to the relaxation time τ, Equation (1).

The limiting high-frequency electrical conductivity κ_∞_ of water, methanol, ethanol, and propanol increases with increasing temperature and passes through a maximum at high temperatures (Figure 2a and Figure 3).

The rate of high-frequency heating of polar solvents at a frequency of 2455 MHz increases in direct proportion to the high-frequency conductivity of the solvents (Figure 9a). The maximum rate of high-frequency heating of aqueous solutions of non-electrolytes occurs at the maximum high-frequency conductivity. (Figure 9b). Microwave radiation is less effective at elevated temperatures in aqueous electrolyte and non-electrolyte solutions.

The specific conductivity of aqueous solutions passes through a maximum with an increase in the concentration of the electrolyte (Figure 10a). In aqueous solutions of lithium, magnesium, calcium, and lanthanum chlorides, the maximum electrical conductivity occurs at a concentration corresponding to the full solvation border—a situation when all water molecules are bound with ions. A further increase in concentration leads to a decrease in the specific electrical conductivity of the solution as a result of the association of ions since there are no longer enough water molecules for the solvation of ions.

The temperature and concentration dependences of the specific conductivity of aqueous solutions of electrolytes are generalized in the coordinates κ/κ_max_−*c*/*c*_max_. In these coordinates, the κ/κ_max_ values fit into a single curve for aqueous solutions of acids, alkalis, and metal salts (Figure 10b), as well as solutions of ionic liquids in acetonitrile, dimethylformamide, and dimethylsulfoxide (Figure 12b).

The specific electrical conductivity of aqueous solutions of inorganic metal salts, as well as non-aqueous solutions of ionic liquids, increases in direct proportion to the limiting high-frequency electrical conductivity of the solvent with increasing temperature (Figure 11 and Figure 13).

For aqueous solutions of inorganic salts, along with Walden’s rule, it is proposed to use the Equation (22), which is valid in the temperature range 0–100 °C.

Equations (26) and (27) were proposed for calculating the distance *d* between particles (ions and molecules) of a solute in a solution and the concentration *c*, at which a certain number of solvent molecules can be located between the ions.

It is assumed that at the maximum of the concentration dependence of the specific electrical conductivity of aqueous solutions of metal salts, one water molecule can be located between the ions. A further increase of concentration leads to the formation of solvate-separated ion pairs. The maximum of the specific electrical conductivity of studied nonaqueous solutions of ionic liquids probably corresponds to the beginning of the formation of contact ion pairs in the solution.

## Figures and Tables

**Table 1:** Static dielectric constant ε_s_, dipole relaxation time τ, and limiting high-frequency electric conductivity κ_∞_ of some polar solvents; t = 25 °C [7].

Solvent	ε_s_	τ, ps	κ_∞_, S/m
Water	78.35	8.25	84.0
Acetonitrile	35.9	4.14	76.8
Acetone	20.7	3.2	57.3
Formamide	109.5	36.9	26.3
Dimethylformamide	37.1	13.1	25.0
Dimethylacetamide	38.6	15.0	22.8
Dimethyl sulfoxide	47.1	19.4	21.5
Propylene carbonate	64.9	42.2	13.6
N-Methylformamide	181	123	13.0
Methanol	32.7	49.6	5.84
Ethanol	24.5	162	1.34
Propanol	20.3	220	0.82
Butanol	17.5	474	0.33

**Table 2:** Dielectric characteristics of 4 M NaCl solution calculated by the Debye equations for different frequencies; *t* = 25 °C [37].

Frequency, GHz	ε′	ε″_d_	ε″_ion_	ε″	δ(ε″_d_), %
3	39.7	3.3	137.9	141.1	154.6
10	36.9	10.0	41.3	51.4	39.2
25	26.7	17.0	16.5	33.5	10.3
50	15.1	15.9	9.2	25.1	7.6
100	8.2	10.1	4.1	14.2	6.4

**Table 3:** High-frequency electrical conductivity κ′ (S/m) at frequency of 2455 MHz for mixtures of water with methanol, ethanol, and propanol at temperatures 10, 25, and 40 °C [56].

Vol. % Alcohol	*t* = 10 °C			*t* = 25 °C			*t* = 40 °C		
	CH_3_OH	C_2_H_5_OH	C_3_H_7_OH	CH_3_OH	C_2_H_5_OH	C_3_H_7_OH	CH_3_OH	C_2_H_5_OH	C_3_H_7_OH
0	2.00	2.00	2.00	1.34	1.34	1.34	0.90	0.90	0.90
10	2.59	3.20	3.60	1.64	2.09	2.39	1.10	1.40	1.67
20	3.08	3.79	4.28	1.96	2.61	3.08	1.31	1.77	2.10
25	3.27	4.11	4.34	2.11	2.88	3.26	1.42	1.99	2.30
30	3.44	4.24	4.31	2.25	3.06	3.36	1.52	2.13	2.43
40	3.65	4.20	4.09	2.49	3.18	3.35	1.71	2.27	2.55
50	3.73	3.95	3.44	2.66	3.18	3.09	1.86	2.36	2.45
60	3.67	3.55	2.87	2.75	3.02	2.73	1.98	2.31	2.27
70	3.50	3.02	2.18	2.74	2.81	2.29	2.03	2.28	2.09
80	3.22	2.37	1.48	2.63	2.43	1.73	2.01	2.15	1.75
90	2.86	1.53	0.82	2.43	1.78	1.05	1.92	1.82	1.19
100	2.44	0.94	0.56	2.15	1.15	0.64	1.74	1.34	0.77

**Table 4:** Limiting HF EC κ_∞_ (S/m) and HF EC at the frequency 2455 MHz κ′ (S/m) of NaCl aqueous solutions in the temperature range 20–50 °C.

*c*, mol/L	*t*, °C							
	20		30		40		50	
	κ_∞_	κ′	κ_∞_	κ′	κ_∞_	κ′	κ_∞_	κ′
0	75.8	1.55	92.7	1.16	111	0.89	130	0.69
0.5	72.6	1.29	88.7	0.96	107	074	125	058
1.0	71.4	1.09	87.2	0.815	105	0.62	123	0.48
2.0	66.9	0.83	82.0	0.62	98.4	0.47	115	0.37
3.0	62.8	0.64	76.8	0.48	92.2	0.36	108	0.28
4.0	59.2	0.50	72.5	0.38	87.1	0.29	102	0.22
5.0	57.6	0.40	70.4	0.30	84.6	0.23	98.2	0.18

**Table 5:** Comparison of calculated and experimental values of concentrations corresponding to the maximum specific conductivity of solutions; ρ—densities of solutions, *N*—number of water molecules bound to ions, δ—divergence of calculated and experimental values of concentrations; *t* = 25 °C.

Electrolyte	κ_max_, S/m [59]	*c*_max_, mol/L [59]	ρ, g/cm^3^ [59]	*c*(H_2_O), mol/L	*N*(H_2_O)	*c*(FSB), mol/L	δ,%
LiCl	18.92	5.33	1.11482	49.88	10	4.99	6.4
MgCl_2_	15.84	2.78	1.19136	50.82	18	2.82	1.4
CaCl_2_	20.46	2.76	1.2252	51.05	18	2.84	2.9
LaCl_3_	17.36	1.62	1.34241	52.48	32	1.64	1.2

**Table 6:** Distance *d* (Å) between particles (molecules and ions) in solutions.

*c*, mol/L	*n*				
	1	2	3	4	5
0.001	118	94.0	82.1	74.6	69.2
0.01	55.0	43.6	38.1	34.6	32.1
0.1	25.5	20.2	17.7	16.1	14.9
1.0	11.8	9.40	8.21	7.46	6.92
5.0	6.92	5.50	4.80	4.36	4.05
10.0	5.50	4.36	3.81	3.46	3.21

## Data Availability

Data supporting reported results can be found in the authors’ publications cited in the references [7,14,15,32,37,38,51,55,56,60,61,62,63,64].
